# Keeping an Eye on PM_2.5_: Satellite Data Reveal Global Picture of Particulate Pollution

**DOI:** 10.1289/ehp.118-a259a

**Published:** 2010-06

**Authors:** Angela Spivey

**Affiliations:** **Angela Spivey** writes from North Carolina about science, medicine, and higher education. She has written for *EHP* since 2001 and is a member of the National Association of Science Writers

Scientists are using satellite observations to provide estimates of air quality with increasing reliability. For the first time researchers have now used satellite data to provide long-term air quality estimates of fine particulate matter (PM_2.5_) that span the globe **[*EHP*** **118:847–855; van Donkelaar et al.]**. The study showed that many developing countries have high long-term levels of PM_2.5_, which is produced by sources such as forest fires, coal-fired power plants, vehicles, and industrial facilities. These particles pose a health concern because of their ability to penetrate deep into the lungs.

The study used satellite data gathered over 6 years, remarkably providing some of the first long-term measurements of air quality for many regions where ground-level sampling stations are few or nonexistent. The scientists combined data gathered from two different NASA satellite instruments with different capabilities—MODIS (Moderate Resolution Imaging Spectroradiometer) and MISR (Multiangle Imaging Spectroradiometer)—to generate a more accurate estimate of PM_2.5_. The satellite data yield a measurement called aerosol optical depth (AOD), which relates to the total amount of aerosol particles in the air between the ground and the satellite. The scientists combined AOD from the two satellites, then applied a chemical-transport model that integrated details about atmospheric structure and chemistry. The authors validated this approach by comparing their estimates to those taken from actual sampling performed at the ground level and found a statistically significant level of agreement.

The estimates showed that 80% of the global population lives in places where concentrations of PM_2.5_ exceed the World Health Organization (WHO) air quality guideline of 10 μg/m^3^. The WHO has set an interim target of 35 μg/m^3^, which is exceeded over central and eastern Asia for 38% and 50% of the population, respectively. (The WHO guideline sets an ultimate goal for national air standards, whereas the interim target is proposed as an incremental reduction that could achieve significant, though not optimal, reductions in pollution-related health effects.) Eastern China showed a very high level of pollution—an estimated annual average of more than 80 μg/m^3^.

The authors state that the methods described and validated by the study could be applied to studies of health effects from exposure to air pollution around the world. This is particularly true for areas where ground-based sampling is lacking, many of which are sites of rapid urbanization, where large populations are exposed to high levels of air pollution. They note that additional work is needed to address issues that may limit the accuracy of the satellite-based estimates, such as non-uniform satellite sampling and the satellites’ inability to retrieve AOD under cloudy conditions.

